# Contemporary management of traumatic cardiac arrest and peri-arrest states: a narrative review

**DOI:** 10.1186/s44158-024-00197-9

**Published:** 2024-09-26

**Authors:** Luca Carenzo, Giulio Calgaro, Marius Rehn, Zane Perkins, Zaffer A. Qasim, Lorenzo Gamberini, Ewoud ter Avest

**Affiliations:** 1 Department of Anesthesia and Intensive Care Medicine, IRCCS Humanitas Research Hospital, Rozzano, Milano, 20089 Italy; 2 Department of Biomedical Sciences, Humanitas University, Pieve Emanuele, Milano, Italy; 3 Pre-Hospital Division, Air Ambulance Department, Oslo University Hospital, Oslo, Norway; 4 Institute of Clinical Medicine, University of Oslo, Oslo, Norway; 5 The Norwegian Air Ambulance Foundation, Oslo, Norway; 6 Centre for Trauma Sciences, Barts and The London School of Medicine and Dentistry, Queen Mary University of London, London, UK; 7 London’s Air Ambulance and Barts Health NHS Trust, Royal London Hospital, London, UK; 8 Department of Emergency Medicine, Perelman School of Medicine at the University of Pennsylvania, Philadelphia, PA USA; 9 Department of Anesthesia, Intensive Care and Prehospital Emergency, Maggiore Hospital Carlo Alberto Pizzardi, Bologna, Italy; 10 Department of Emergency Medicine, University Medical Centre Groningen, University of Groningen, Groningen, the Netherlands

**Keywords:** Traumatic cardiac arrest, Trauma, Thoracotomy, Clamshell, REBOA, Hypovolaemia, Exsanguinating, Tension pneumothorax, Cardiac tamponade, Hypoxia

## Abstract

Trauma is a leading cause of death and disability worldwide across all age groups, with traumatic cardiac arrest (TCA) presenting a significant economic and societal burden due to the loss of productive life years. Despite TCA’s high mortality rate, recent evidence indicates that survival with good and moderate neurological recovery is possible. Successful resuscitation in TCA depends on the immediate and simultaneous treatment of reversible causes according to pre-established algorithms. The HOTT protocol, addressing hypovolaemia, oxygenation (hypoxia), tension pneumothorax, and cardiac tamponade, forms the foundation of TCA management. Advanced interventions, such as resuscitative thoracotomy and resuscitative endovascular balloon occlusion of the aorta (REBOA), further enhance treatment. Contemporary approaches also consider metabolic factors (e.g. hyperkalaemia, calcium imbalances) and hemostatic resuscitation. This narrative review explores the advanced management of TCA and peri-arrest states, discussing the epidemiology and pathophysiology of peri-arrest and TCA. It integrates classic TCA management strategies with the latest evidence and practical applications.

## Background

Trauma is a major cause of death and disability worldwide in all age groups [1]. Traumatic cardiac arrest (TCA) is the common term used to describe circulatory arrest following major trauma. According to the EuReCa TWO European-wide study, TCAs account for approximately 4% of all out-of-hospital cardiac arrests [2]. However, low- and middle-income countries (LMICs) suffer disproportionately from injuries, which are among the top causes of death, particularly in younger people [3]. While a comprehensive global overview of TCA epidemiology is lacking, existing data likely underestimates the full extent of the global issue. Traumatic cardiac arrest (TCA) has a high mortality rate, ranging from 92.3 to 100%, depending on whether prehospital deaths are included or on the development of the trauma system [4–6]. However, recent epidemiological data have demonstrated that survival is possible, with a 10.6% 30-day survival rate reported in a recent Swedish epidemiological study [7]. Among those that survive however, good and moderate neurological recoveries are frequently reported. In a systematic review on outcomes following TCA, 45.8% of survivors showed good or moderate neurological recovery, 29.0% experienced severe neurological disability or were in a vegetative state, while 25.2% had missing neurological outcomes [8]. TCA predominantly affects relatively young, mostly male patients, with a median age of 38 years. Most of these patients are previously healthy, with 60.5% having no significant prior health issues. Age and previous health status support the recovery potential of the patients but also underline the economic and societal burden of productive life years lost in the many cases of unsalvageable TCA [7–9]. The mechanism of injury is blunt in approximately two-thirds of TCA cases; however, the epidemiology can vary depending on geographical areas, with penetrating trauma increasing in metropolitan areas or regions where firearms are widely available [10, 11]. A contemporary approach to the problem must consider that TCA is not a unique and stationary condition but rather an umbrella term that encompasses a spectrum of physiological conditions that are clinically indistinguishable resulting in an unconscious patient without palpable pulses [12]. The underlying physiology can vary from low-output state in trauma (LOST) to no-output states in trauma (NOST) [13]. LOST can be described by an unconscious patient, not breathing and with no palpable pulse (hence a classic definition of cardiac arrest) but still presenting organized electrical activity, and/or some visible cardiac motion on focused ultrasound [14]. Conversely, NOST is associated with bradycardia or agonal electrical rhythms and likely cardiac standstill at ultrasound. In a large epidemiological study, patients with LOST had an 8.4% survival rate, while those with NOST had a 0% survival rate [13]. Peri-arrest refers to the moment just minutes or seconds before circulatory arrest. It is often identified by extreme low-flow states that clinically can be identified by pallor, sweating, cold, clammy extremities (increased sympathetic activity), impaired consciousness or syncope (reduced cerebral blood flow), and hypotension [15]. Identification and action in this period are particularly clinically relevant as the application of intervention in the peri-arrest period yields the maximum success rate of benefit for successful patient outcomes. The aim of this paper is to provide a narrative review on the contemporary management strategies for traumatic cardiac arrest and peri-arrest states, highlighting the importance of early identification and intervention to improve patient outcomes.

## Pathophysiological considerations and resulting treatment consequences

The causes of TCA and the preceding peri-arrest states typically stem from hypoxia (13%), hypovolaemia (48%), and/or obstructive physiology (caused by cardiac tamponade 10% and pneumothorax 13%) [16]. Possible causes of hypoxia that can cause TCA following trauma are impact brain apnoea (the phenomenon of apnoea following traumatic brain injury), damage to the airway (obstruction), or damage to the lungs including pneumothorax [17]. Tension physiology might mimic hypovolaemia, both leading to a decrease in venous return and consequently stroke volume. Hypovolemic TCA is characterized by inadequate cardiac output due to reduced left ventricular volume resulting from either hypovolaemia or restricted ventricular filling. The pathophysiology of exsanguination shows two distinct phases: an initial drop in blood pressure and cardiac output, with coronary flow relatively maintained until reaching a critical blood pressure threshold, at which point coronary flow ceases, leading to asystole. The pathophysiological rationale for aggressively applying the interventions described in this review is to re-establish effective coronary blood flow, with the aim of obtaining ROSC [18].

Collectively, hypoxia, hypovolaemia, tension pneumothorax, and cardiac tamponade are referred to as addressable causes, which may be reversible or non-reversible. In clinical practice, it is often challenging to determine at presentation whether the causes of TCA are irreversible. Therefore, it is generally accepted to focus on addressing a core set of potentially reversible causes before considering the termination of resuscitative efforts. Reversible addressable factors, which may restore oxygen delivery, are often recalled using the acronym HOTT, representing hypovolaemia, oxygenation, and tension/tamponade [19]. These reversible factors can occur concurrently and may exacerbate each other. Hence, it is imperative to address them simultaneously rather than sequentially. Moreover, in a contemporary approach, HOTT is not sufficient; metabolic derangements should also be considered, especially hyperkalaemia. The resulting metabolic derangements are often severe following TCA due to exsanguination but much less so for obstructive causes of TCA. This disparity explains the much better outcomes observed for tamponade and tension pneumothorax TCA compared to exsanguination TCA, underscoring the importance of always looking for and addressing obstructive causes, although they are less common.

Under euvolemic conditions, external cardiac massage, when executed well, provides only 25–30% of baseline cardiac output and approximately 10% of the baseline cerebral and coronary perfusion [20]. Animal studies on both hemorrhagic and obstructive shock have found that external chest compressions do not offer additional benefit compared to appropriate treatment of reversible causes [20–22]. Lack of effect of external compressions is explained by reduced coronary flow resulting from hypovolaemia or obstruction. Key to return of spontaneous circulation (ROSC) and to maintain an efficient cardiac activity is a sufficient coronary perfusion. Coronary perfusion pressure (CPP) serves as an indicator of myocardial perfusion (and hence oxygen delivery), with a threshold of 15 mmHg considered necessary to obtain ROSC and is dependent on the pressure gradient between the aortic diastolic blood pressure and the left ventricular end-diastolic pressure. Whilst normally, in patients who are not hypovolemic, external chest compressions increase diastolic blood pressures, this is not the case in patients with a tamponade or hypovolaemia [23, 24].

Key message is that prioritizing immediate treatment of reversible factors aiming at re-establishing effective coronary blood flow should take priority over administering chest compressions [25]. Chest compressions can be initiated before addressing reversible causes if there are enough (human) resources in the resuscitation team to provide both high-quality chest compressions while not hindering immediate management of potentially reversible causes. Nevertheless, if there is uncertainty or in specific subcategories of TCA, high-quality chest compressions should be initiated. These scenarios encompass potential unclear medical or cardiac origins of arrest, as well as TCA stemming from non-hypovolemic, non-obstructive causes, such as isolated traumatic brain injury (TBI), cardiac contusion, asphyxiation, and drowning [9].

### Blunt vs. penetrating trauma

Penetrating and blunt trauma have different pathophysiology and outcomes. Blunt trauma results from impacts with dull objects, often causing multiple injuries and often involving the central nervous system. In contrast, penetrating trauma, caused by bullets or stab wounds, usually leads to single-system injuries, with neurological damage being rare. Penetrating trauma is classified by velocity: low (knives), medium, and high (gunshots) [26]. Patients with penetrating injuries are 3.5 times more likely to suffer named vascular injuries and experience higher rates of TCA due to rapid deterioration. Despite this, penetrating trauma has a higher survival rate (10.6%) compared to blunt trauma (2.3%) because vascular injuries are often treatable with resuscitation techniques. Understanding these differences improves patient management and treatment [27, 28].

## General principles of management

**Fig. 1:**
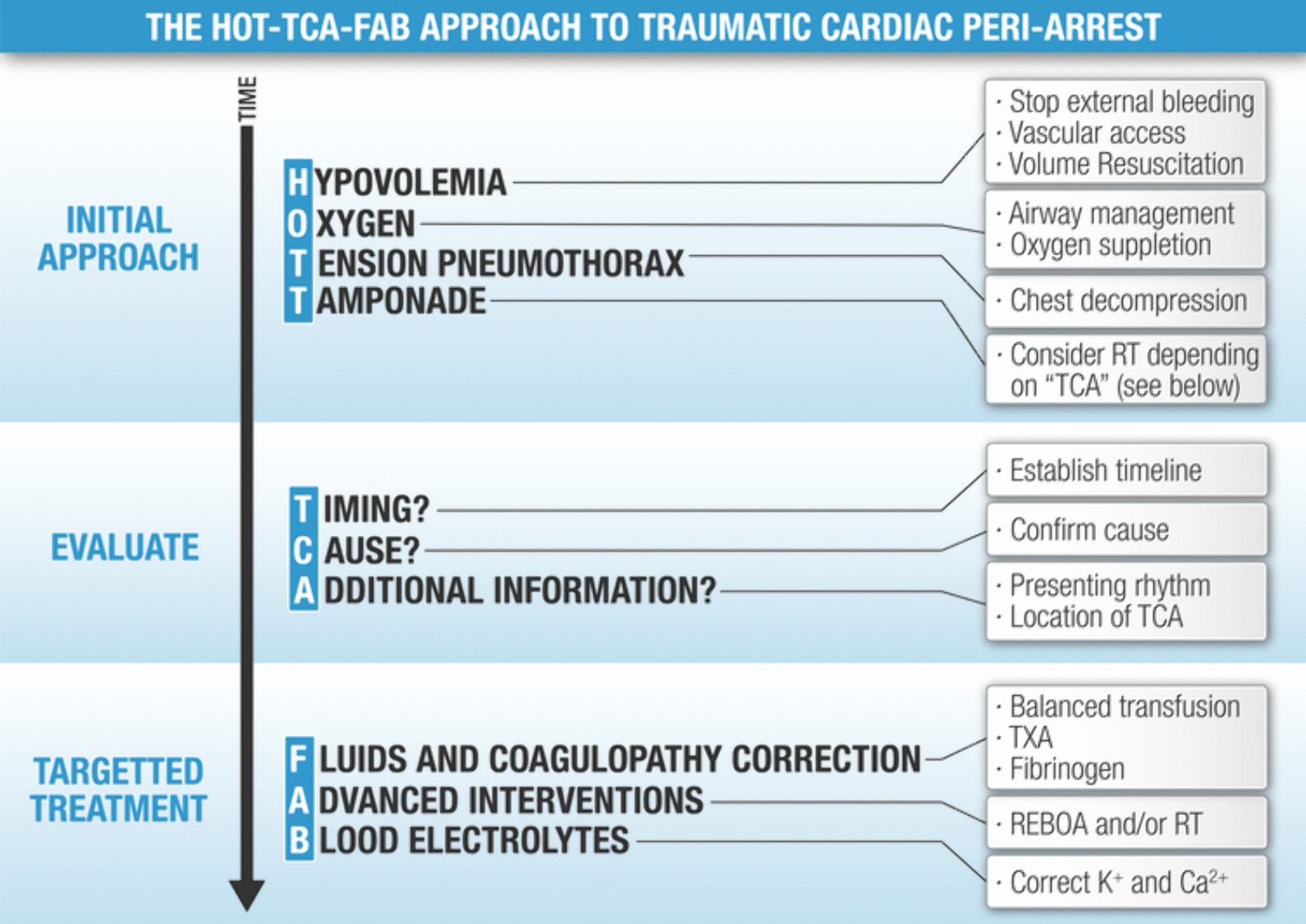
Proposed mnemonic for the pragmatic approach to a traumatic cardiac arrest

Given limited time to establish the exact causes of TCA, using “immediate action drills” that rapidly address the most prevalent potentially survivable aetiologies is preferred over performing selective actions based on a diagnostic process [29], especially given that diagnosing life-threatening injuries in trauma is difficult and studies suggest clinical examination yields only a moderate accuracy [30]. This also spares mental bandwidth in a stressful situation, allowing the clinician to make complex decisions (i.e. performance of advanced interventions). Key aspects of TCA management are the rapid and simultaneous delivery of multiple interventions to address all the potential reversible causes [31]. Interventions can be divided into mandatory and optional interventions. Where the former need to be performed in all patients with TCA, the latter are suitable only for selected patients and/or selected situations. It is worth remembering that optional interventions are not necessarily performed after mandatory. It is difficult to attribute any improvement in survival to one single intervention, and perhaps this is best considered as a bundle of care for patients in TCA [13]. Figure 1 presents the HOTT-TCA-FAB mnemonic covering the main aspects of TCA management, a suggested order for the clinician to keep things simple in an often very hectic, resource-consuming, and time-dependent situation.

Finally, it is important to remember that the overall philosophy of improving TCA outcomes lies in delivering appropriate interventions at the right time and place. Ideally, due to the time dependency of therapeutic actions, state-of-the art treatment should be delivered at the site of arrest (either prehospitally or in hospital) by trained clinicians as this has been shown to reduce TCA mortality [4]. While all of the interventions discussed have been described in both prehospital and in-hospital settings, a pragmatic approach will depend on the local system in place. This decision must take into account the technical capabilities, available skills, training, governance, and human resources [32]. Consequently, a one-size-fits-all solution is rarely feasible, and a thorough understanding of the local prehospital, in-hospital, and ancillary services (i.e. blood bank) capacities is essential to determine the most appropriate setting for each intervention described.

## Basic interventions: treat hypovolaemia, oxygenation, and tension/tamponade.

Interventions ideally should be simultaneous, but in case it is not possible, the priority is to re-establish circulation.

### Haemorrhage control

Direct pressure remains the primary treatment approach to control external haemorrhage. Large wounds should be packed using hemostatic gauzes applied directly to the source, packing tightly until the wound is filled with gauze to provide sufficient pressure [33]. A tourniquet can be applied if anatomically feasible. Once applied, the tourniquet should not be loosened until definitive care options are available. It is essential to regularly re-evaluate both the wound and the tourniquet, especially after any movement by the patient. If effective haemostasis is not achieved, a second tourniquet can be applied 2–3 inch above the original one without removing the latter [34]. If noncompressible bleeding is suspected in the context of traumatic peri-arrest, the top priority should be to quickly transport the patient to a facility where haemorrhage control can be achieved, depending on the availability of advanced interventions. Pelvic ring injuries are present in a significant number of patients after blunt trauma and may result in severe hemorrhagic shock [35]. Consequently, effective management of pelvic fractures should include strategies to control associated haemorrhage. If the patient is already in TCA, there is no time to assess whether the pelvis is injured. A pelvic binder can be applied to any blunt traumatic cardiac arrest with suspicion of pelvic injuries; meanwhile, REBOA (resuscitative endovascular balloon occlusion of the aorta) or preperitoneal packing are evaluated or performed. In TCA, the patient may have little or no active bleeding initially, but bleeding may resume once appropriate management, such as aggressive volume restoration, is initiated, and this is why including a stop-the-bleed approach is part of TCA basic management [36].

### Vascular access

A crucial aspect of TCA management is establishing rapid and high-flow vascular access to allow fluid resuscitation [37]. Peripheral intravenous (PIV) catheters are commonly used, with classic trauma literature recommending early placement of multiple large bore (14G) PIVs. However, obtaining PIV access can be challenging in exsanguinating TCA patients who may present with no visible or palpable peripheral vessels. The use of ultrasound-guided PIV may be time-consuming in such cases. Intraosseous access (IO) has been suggested as the fastest method in hypotensive trauma patients, with higher success rates compared to PIV and central venous catheters (CVC), as per a retrospective review [38, 39]. However, the flow rate of IO (50 ml/min) is significantly lower than that of PIV or large-caliber CVC (such as rapid infusion catheters or “trauma lines” also commonly called “introducers” or “sheaths”) [40, 41]. As rapid volume expansion with blood products is a key procedure in TCA resuscitation, CVC may therefore be the preferred access for the optimal management of exsanguinating cases, especially when large bore peripheral IV access cannot be readily obtained [42].

Trauma-dedicated CVCs (rapid infusion catheters) are typically large bore (8.5 or 9 Fr) and short in length (i.e. 10 cm), allowing for maximum flow rate and minimal resistance [43]. The choice of insertion site and technique often depends on clinical habits and operator expertise, with the right subclavian vein being commonly utilized, as it is, in the authors’ experience, the vessel less prone to collapse in extreme hypovolemic conditions. Other described strategies include accessing the femoral or the jugular vessels [44]. When considering the insertion site, the treating clinician must consider however that supradiaphragmatic venous access is to be preferred in noncompressible torso haemorrhage, as this will allow infusions to effectively reach the heart regardless of any torso damage present [45]. On the other hand, when patients have subdiaphragmatic injuries requiring damage control surgery (with retrohepatic inferior vena cava clamping or the Pringle manoeuvre), a venous catheter in the femoral vein may become ineffective for volume replacement and medication administration. A dedicated vascular access operator, often an experienced anaesthesiologist or an emergency physician, can be part of the trauma team and is prepared with a sterile setup, an open kit, and a needle in hand. Once the TCA patient is transferred to the trauma bay bed, the vascular access operator promptly proceeds with cannulation. The catheter is then connected to a pre-primed rapid infuser, which combines a filter, pressure or roller pump, and heat exchanger to deliver warmed fluids at rates of up to 750 mL/min. This system allows for the continuous infusion of cold blood directly from blood bank packs. The prehospital environment should not limit the administration of massive transfusions, although clinicians must be aware of the logistical challenges. High-performance, battery-operated blood heaters are commercially available and, when used in conjunction with a simple pressure bag, standard operating procedures, and team training, can significantly improve process outcomes in this setting [46, 47]. Arterial cannulation is not an immediate priority and should be deferred until other essential procedures have been performed, unless a clear trajectory towards resuscitative endovascular balloon occlusion of the aorta (REBOA) has been determined or decision to position an early femoral access (EFA) has been made (see below).

### Fluid resuscitation

A patient in TCA caused by hypovolaemia is unlikely to achieve ROSC unless haemorrhage control is performed whilst at the same time the intravascular volume is replaced [19, 48]. Balanced transfusions are currently the most common fluid resuscitation strategy for patients with severe bleeding, including TCA, with crystalloids and colloids used only when the former are not (readily) available [49, 50]. This approach involves continuously administering packed red blood cells, fresh-frozen plasma, and platelets in a 1:1:1 ratio [51]. The potential therapeutic and logistical advantages of (reconstituted) low titre whole blood is currently under investigation in a civilian setting [52]. It is challenging to guide resuscitation using viscoelastic tests as these assays are ex vivo analyses of dynamic in vivo systems; consequently, relying on fixed ratios for administering blood components is a rational approach during the initial phase of TCA resuscitation [53]. Details of fluid resuscitation in trauma should be defined in a local massive transfusion protocol (MTP) that is not only for clinical guidance but also to facilitate rapid delivery of large volumes of blood products [50]. The longer it takes to activate the MTP, the worse the patient outcome [54]. Ideally, the hospital MTP should be activated by the prehospital system [55] or as a minimum blood product should be in the ED available before patient arrival. Prehospital administration of blood components is also an attractive solution being investigated in the optimal management of prehospital TCA [56].

### Airway management and oxygenation

As hypoxia may be a precipitating cause or contributing factor to TCA, it is important to ensure early into the resuscitation attempt that a patent airway is present and that oxygenation is optimized aiming at normoxia and normocapnia. During the initial phase of the resuscitation, basic airway manoeuvres and simple (two-handed) bag valve mask ventilation or ventilation through supraglottic airway devices may serve this purpose. Therefore, time-consuming and resource-intensive airway-securing procedures, such as endotracheal intubation, should be deliberately de-emphasized or postponed during the very early phase of resuscitation, provided an open airway is present or can be quickly established noninvasively [57]. Although this review focuses primarily on hemorrhagic and/or obstructive TCA, specific aetiologies of TCA can benefit from early advanced airway management such as TCA caused by asphyxia, drowning, and isolated traumatic brain injury.

### Treatment of obstructive shock pathology

In TCA patients, clinicians should not waste time by trying to diagnose pneumothoraces either clinically or with ultrasound. Tension pneumothorax should be systematically excluded by performing bilateral chest decompression [14]. This can be achieved by needle decompression or, when the right skills and materials are present, by performing finger thoracostomies. While the latest treatment recommendations from advanced trauma life support (ATLS) suggest placing the needle in the 4th/5th intercostal space just anterior to the mid-axillary line, the evidence supporting this recommendation is weak, and the available data on the topic are highly heterogeneous (ATLS). Additionally, Azizi et al. demonstrated that in overweight and obese patients, the chest wall is thicker in the 4th/5th intercostal space in the anterior axillary line (ICS 4/5-AAL) than in the 2nd intercostal space in the midclavicular line (ICS2-MCL), leading to theoretically higher success rates of needle decompression of tension pneumothorax in ICS2-MCL compared to ICS 4/5-AAL [58]. Even with placement in the 2nd ICS however, a certain percentage of needle thoracostomies do not reach the thoracic cavity or dislodge, kink, or clot after placement. Therefore, when possible, performing finger thoracostomies bilaterally is preferred over needle decompression [59]. Finger thoracostomy is an easy technique that should also be applied prehospitally [60]. Finger thoracostomies are associated with a higher rate of pleural cavity access and better rate of vital signs improvement, although overall mortality remained unaffected [61]. Obstructive shock from a cardiac tamponade requires pericardial decompression via resuscitative thoracotomy (RT). Cardiac tamponade in trauma is most commonly caused by penetrating injuries to the “cardiac box”, typically from a stab wound to the chest just left of the sternum. It can also occur, though less frequently, from blunt trauma involving a strong impact to the chest, which may be accompanied by a sternal fracture. Depending on the setting (prehospital vs. in-hospital), available human resources, operator confidence, and technology availability, point-of-care ultrasound (POCUS) can assist in diagnosing cardiac tamponade as traditional clinical signs such as hypotension, distended neck veins, and muffled heart sounds will not be reliable during TCA or peri-arrest. However, it is crucial to emphasize that POCUS should not delay the decision to proceed with therapeutic interventions such as RT. In the case of pericardial tamponade, RT should be the first intervention and should be done immediately taking precedence over all other interventions. No other action such as airway management or chest compression is of any help until the pericardium has been decompressed. Survival is extremely time critical. Details about this procedure will be presented below including the possible application of ultrasound in the diagnostic process of tamponade.

## Advanced interventions

Advanced interventions such as RT and REBOA should only be considered in selected patients that meet specific criteria. RT and/or REBOA should only be performed in combination with aggressive volume resuscitation, external or internal cardiac massage, low-dose titrated vasopressors, and in general all the mandatory interventions described above [12].

### Resuscitative thoracotomy

RT is an invasive life-saving procedure, serving multiple purposes including treating cardiac tamponade, controlling haemorrhage, managing sub-diaphragmatic haemorrhage by proximal aortic control, and providing internal cardiac massage to better improve diastolic blood flow and increase the chances of ROSC [28, 62, 63]. It is important to underline that the role of RT is different depending on the underlying cause of TCA. In case of cardiac tamponade, RT is the treatment of choice and should be performed swiftly preceding all other interventions. In the absence of pericardial tamponade causing TCA, RT can be considered integrating information regarding timing, mechanism, and signs of life (SOL). In cases of TCA due to penetrating injuries, emergency thoracotomy is indicated for patients who are pulseless and have received less than 10 min of CPR after the injury. For TCA resulting from blunt trauma, RT can be considered for patients who experience arrest upon arrival or are pulseless but show SOL, such as pupillary response, spontaneous ventilation, or cardiac electrical activity. RT is generally contraindicated if definite loss of cardiac output is present for more than 10–15 min [25, 64–66].

Significant differences in outcomes exist following RT for penetrating versus blunt mechanisms. A recent nationwide analysis in the United States (US). examined outcomes after RT based on age, SOL, and injury mechanism. The overall survival to discharge rate was 19.9%: 26.0% in penetrating and 7.6% in blunt trauma. This can be explained by the different nature of injury: blunt trauma often results in severe injuries in various body regions, making the chances of survival following RT in TCA cases following blunt trauma small [67]. RT showed the highest survival rates in patients under 60 years old who presented with SOL after penetrating trauma. None of the patients with blunt trauma and no SOL survived [28].

**Fig. 2:**
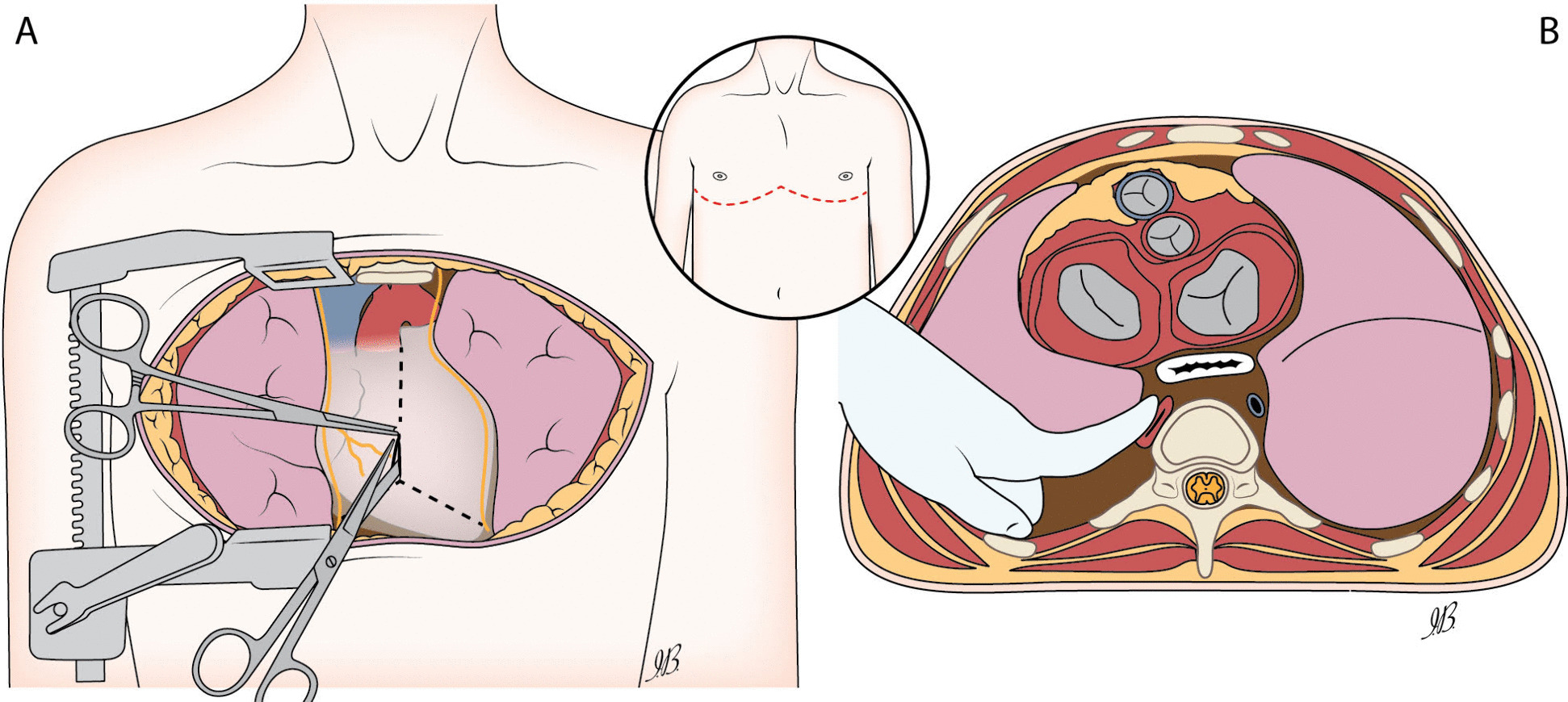
Resuscitative thoracotomy. **A** Front view of clamshell incision and pericardial opening with inverted “T” incision. **B** Axial view of the chest with operator’s hand performing manual aortic occlusion

The key aspect of RT is decision-making and timing of execution [66]. RT should be performed, when and where needed, without delay within the above-mentioned time limits by any trained member of the trauma team regardless of the base specialty (surgery, anaesthesia, emergency medicine, etc.) depending on the local availability and structure of the trauma team. Surgeon vs non-surgeon operators might adopt different techniques, but a basic RT is composed of the following minimum actions: clamshell approach, pericardial opening and heart delivery, aortic compression, and internal cardiac massage (Fig. 2) [63, 65].

Decision-making can be enhanced using ultrasound, which helps confirm tamponade and gain information on signs of life, particularly in low-volume centres that may struggle with the decision-making process, as well as in centres with high proficiency in ultrasound use and round-the-clock availability of highly skilled staff [68, 69]. However, in cases of doubt, especially with central penetrating trauma, it is essential to minimize diagnostic time and proceed swiftly with the procedure. Left anterolateral approach, wound repair, stitching the heart, or lung haemostasis are more complex operations and are usually reserved for more experienced operators [65]. The technique should be straightforward, swift, and provide good chest exposure, and the equipment should be minimal and easily recognizable [64]. Other interventions, such as vascular access, intubation, and ventilation, should be carried out by other team members simultaneously without delaying the RT. Surgical draping and sterility are not essential [64].

If, after opening the chest, the heart is not contracting (whilst fluid resuscitation, aortic compression, and internal massage are ongoing), it can be stimulated by flicking with a finger. Ventricular fibrillation may occur during RT, and defibrillation with internal paddles can be initiated with 10-J energy [65]. If internal paddles are not available, conventional defibrillation is carried out by removing the rib spreader, closing the chest, applying electrodes, and starting defibrillation. It is crucial during internal massage to compress the aorta against the spinal column to maximize cerebral and coronary perfusion until ROSC is eventually achieved or termination of resuscitation is considered.

### Resuscitative endovascular balloon occlusion of the aorta (REBOA)

REBOA is a technique that involves inserting a percutaneous balloon into the aorta via the common femoral artery to rapidly control haemorrhage and support perfusion to the heart and brain (increasing cardiac afterload and proximal aortic pressure, improving coronary and cerebral perfusion) until definitive surgical or endovascular control of bleeding can be achieved. It should be stressed that it is not a definitive treatment, but a temporizing measure as distal perfusion should be restored as soon as possible to minimize the deleterious effects of distal ischaemia [70]. TCA patients may require complete aortic occlusion until ROSC is obtained. As soon as physiologically feasible, partial occlusion (pREBOA) should be obtained thereafter [71].

From determining the correct site of balloon placement, the aorta is divided into three anatomical regions: zone 1 between the left subclavian artery and celiac trunk, zone 2 between the celiac trunk and lowest renal arteries, and zone 3 between the lowest renal arteries and aortic bifurcation [72].

**Fig. 3:**
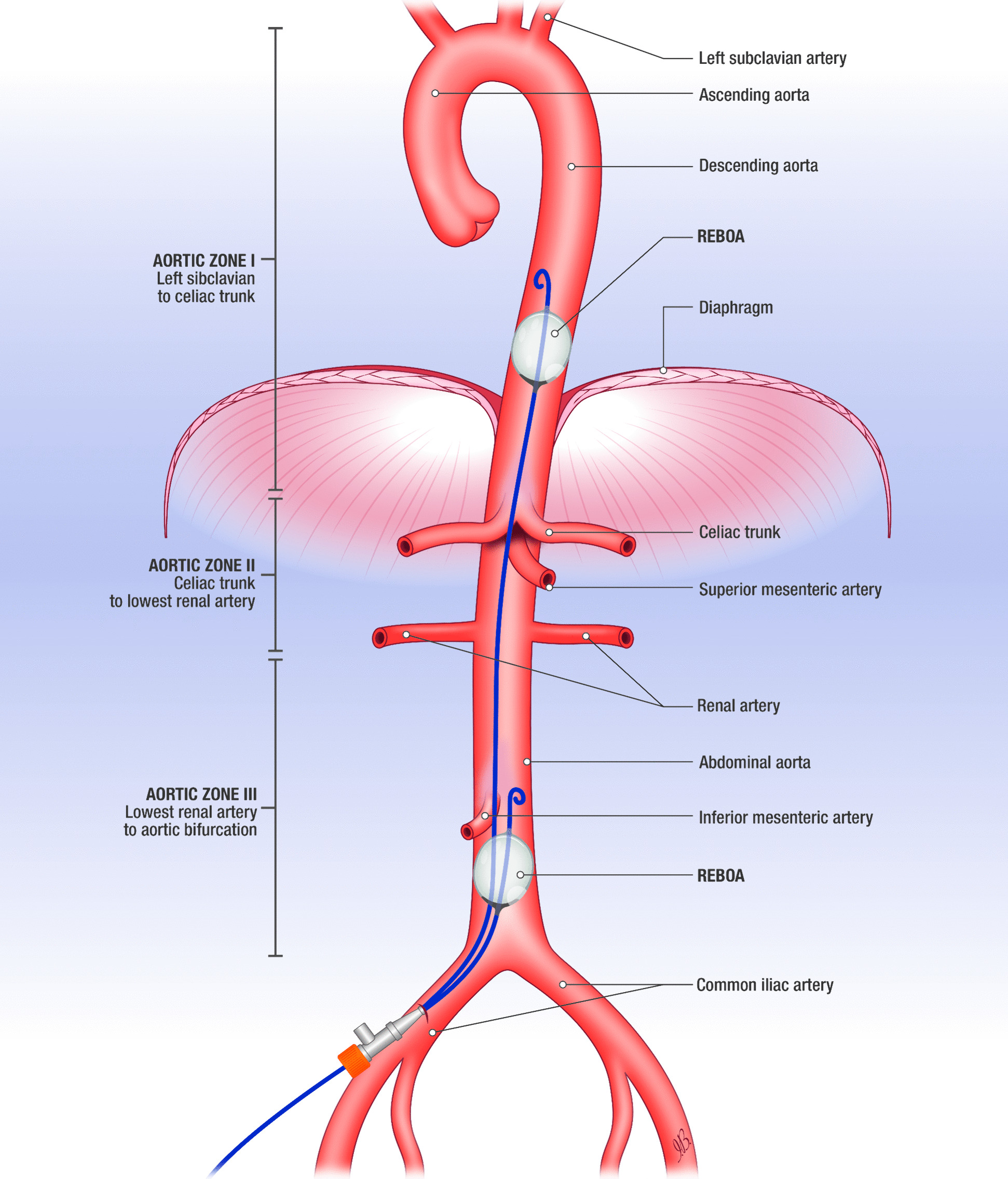
Resuscitative endovascular balloon of the aorta (REBOA)

Inflating the balloon in zone 1 helps reduce bleeding below the diaphragm (abdominal or pelvic injuries), zone 3 is used to reduce haemorrhage from the pelvis and lower extremities, while placement in zone 2 should be avoided [73]. It should be stressed that even if the balloon is well-positioned, there is a risk of distal ischaemic damage (kidney injury, mesenteric ischaemia, paraplegia, multi-organ failure) and reperfusion injury [74]. Placement in either zone increases afterload (and thereby diastolic coronary perfusion), but this increase is higher for zone 1 placement, the preferred site for patients in TCA (Fig. 3).

The time taken to insert the device may be crucial for the patient’s survival. In cases of difficult or unsuccessful cannulation, conversion to RT must be considered for haemorrhage control. No hard timelines can be provided when this should occur, but as survival decreases rapidly with each minute in arrest, in the author’s experience, this decision is generally made within 5 min.

The survival benefit of REBOA compared to non-REBOA in cases of severe abdominal-pelvic haemorrhage, as well as between REBOA and RT in cardiac arrest due to haemorrhage, is a subject of controversy [73]. However, despite the low quality of evidence, an Italian group found a difference in favour of REBOA over RT. Moreover, a comparison between RT with internal cardiac massage and closed chest compression showed end-tidal CO2 values to be similar between groups, suggesting that RT for the sole purpose of resuscitative cardiac compression and aortic occlusion may not be necessary with the advent of REBOA [75].

A recent systematic review showed considerable variation in contraindications to REBOA, with 20% of published algorithms not providing any specific contraindications [76]. The remaining 80% broadly mentioned chest trauma as a contraindication, defined variably as major bleeding “proximal to the left subclavian artery”, “severe blunt chest injury”, “major thoracic vascular injury”, or “thoracic haemorrhage”. Despite the lack of strong evidence, the associated morbidity of opening the chest to cross-clamp the aorta makes REBOA an attractive procedural option compared to RT [77]. However, in patients who experience TCA secondary to penetrating thoracic trauma, RT should still be preferred as REBOA offers no ability for direct injury repair and occluding the descending aorta may worsen proximal haemorrhage.

### Early femoral access (EFA)

Early femoral artery access refers to the procedure of quickly obtaining vascular access to the femoral artery, usually through percutaneous techniques or surgical cutdown, in the early phases of haemorrhagic and pre-arrest patient management. This access is typically achieved by positioning a sheath that can be initially smaller in diameter to facilitate invasive blood pressure monitoring and can be easily upscaled to a larger sheath capable of accommodating a REBOA catheter. Early access is critical as it is usually technically easier to position the sheath when perfusion is present rather than during cardiac arrest. EFA allows for rapid deployment of life-saving interventions without impeding resuscitation efforts and is associated with shorter times to haemostasis in severely injured patients [78]. A recent consensus suggests that EFA should be considered for all trauma patients with an initial systolic blood pressure (SBP) < 60 mm Hg, regardless of their response to fluid therapy. It is also considered for those with an initial SBP < 90 mm Hg who do not respond to fluid or blood administration [79].

### Role of vasopressors

Generally, there is no place for fixed-dose interval administration of vasopressors in TCA [80]. Various retrospective trials have reported an association with a lower chance of neurologically intact survival (although selection bias may have influenced these findings). Exemptions are TCA with an hypoxic- or CNS aetiology, where normal resuscitation doses of adrenaline may be used. Based on the author’s experience, for patients experiencing TCA due to hypovolemic or tamponade causes, a low-dose administration (100 mcg bolus) may be considered as an adjunct to resuscitation, particularly to sustain cardiac activity following ROSC.

## Metabolic, haemostatic, and post-ROSC care of TCA

These interventions are not traditionally included in the treatment of reversible causes; however, they might play a role in both preventing traumatic cardiac arrest (TCA) from a peri-arrest state, facilitating ROSC (hyperkalaemia), and managing the patient following ROSC. The optimal timing depends on the patient’s clinical condition, the clinician judgment, and resources available.

### Tranexamic acid

Tranexamic acid (TXA) is now an essential component of the multimodal treatment approach for major trauma patients [81].

One of the most crucial aspects in TXA administration is the timing. Early treatment (≤ 1 h from injury) significantly reduced the risk of death due to bleeding, and as such TXA should be given as early as possible following injury [82].

The use of TXA in traumatic cardiac arrest (TCA) remains under-investigated. A recent study in Japan suggested that its use was associated with a higher rate of ROSC [83]. TXA should continue to be used as part of a multimodal approach to treating TCA. However, its administration should never take precedence over other, more important interventions. Compared to the CRASH-2 regimen of a 1-g bolus followed by a 1-g infusion over 8 h, a 2-g bolus is now being used in some centres; it has been proven as effective and is likely more practical in its delivery [84].

### Potassium correction

Early hyperkalaemia following severe haemorrhagic shock has been reported in clinical studies [85]. Perkins et al. found that 29% of patients had hyperkalaemia without acidosis, and all patients with hyperkalaemia died [86]. In a swine model, Rocha Filho et al. observed a significant increase in potassium levels during the early haemorrhagic phase [87]. They also found that hyperkalaemia occurs early in haemorrhagic shock after liver trauma. Massive transfusions may also contribute to hyperkalaemia with potassium leakage from stored blood, and the release of intracellular potassium into extracellular spaces due to haemolysis is triggered by mechanical agitation of red blood cells during transfusion. Animal studies suggest that aggressive potassium correction improves the probability of ROSC [88].

Any hyperkalaemia should be aggressively treated to re-establish normal serum potassium concentrations. Feasible medical management of hyperkalaemia during TCA includes protecting the heart (by supplementing calcium) and reversing cellular potassium efflux with insulin boluses. During TCA, administering insulin as a bolus is the most effective way, giving 10 units soluble insulin and 25-g glucose IV by rapid injection [25]. Monitor potassium levels closely as multiple boluses or a continued infusion are likely to be needed. A pragmatic approach is the recently published hyperkalaemia protocol from the Royal London Hospital Trauma Anaesthesia Group. An infusion of 50 IU of fast-acting insulin in 50 mL of 50% glucose is started at a rate of 2–3 mL/h and titrated to serum potassium. Rates of up to 200 mL/h are necessary to limit potassium levels in patients receiving the highest rates of blood product infusion, successfully resuscitated from traumatic cardiac arrest, or undergoing open or endovascular aortic occlusion [89].

### Calcium administration

Hypocalcaemia is frequently observed in patients with different types of trauma. The normal range of ionized calcium in humans is 1.15–1.3 mM/L. Studies have demonstrated that mild hypocalcaemia (0.9–1.15 mM/L) is present in 64% of trauma patients upon hospital admission. Severe hypocalcaemia (< 0.9 mM/L) was found in 10% of trauma patients and is an independent risk factor for coagulopathy and massive transfusion [90, 91]. Hypocalcaemia can result from consumption as a clotting cofactor, intracellular flux of calcium due to ischaemia and reperfusion, due to loss of blood (and calcium ions), and from the citrate administered with blood transfusions [92]. Efforts should therefore be made to limit the amount of citrate infused into a patient in haemorrhagic shock while simultaneously addressing the induced hypocalcaemia [93]. TCA patients can benefit from serial blood gasses to monitor and treat accordingly calcium imbalances, and if Ca2 + measurement is not readily available such as in the prehospital setting, empirical treatment can be considered; empiric administration outside of TCA may be harmful as both hypo and hypercalcaemia are associated with poorer outcome [94].

### Post-resuscitation care

Post-ROSC care potentially impacts prognosis significantly. For patients who obtain ROSC after TCA, the management after ROSC is guided by the underlying cause. Nevertheless, some interventions should be considered in all patients. These include transporting the patient to a major trauma centre (for prehospital TCA), administering appropriate sedation, analgesia, and neuromuscular blockade if needed, and maintaining normothermia during transport. The choice of sedative agent should prioritize those with minimal impact on cardiovascular stability, such as ketamine or etomidate, depending on local availability and practice. Very small additive boluses of fentanyl can also be used to achieve the desired analgesic effect.

Following the HOTT protocol, we can highlight selected post-ROSC care for each reversible cause: hypovolaemia and haemostatic management. Following the initial phase of fixed-ratio aggressive volume expansion, a tailored approach towards volume and haemostatic resuscitation can be considered. TCA patients often will be profoundly coagulopathic. Reduced levels of fibrinogen are prevalent, and fresh-frozen plasma has a low content of fibrinogen. To address this issue, the use of fibrinogen concentrate could be considered as an additional component to the balanced transfusion [95, 96]. Pragmatic administration of fibrinogen to all trauma patients receiving a transfusion has not been shown to improve all-cause 28-day mortality [97]. The use of viscoelastic tests combined with an institutional protocol could help to goal-direct haemostatic resuscitation allowing precise administration of needed components.

- Oxygenation: Advanced airway post-ROSC is desirable, and orotracheal intubation is optimal if provider expertise allows to safely do so. It is crucial to avoid hyperventilation, as it can increase intrathoracic pressure, reduce venous return to the heart, and impair coronary and cerebral perfusion. Post-return of spontaneous circulation (ROSC), ventilation should be titrated to achieve normal oxygen and carbon dioxide levels, adopting a lung-protective strategy with low tidal volumes and appropriate positive end-expiratory pressure [98].
- Tension pneumothorax: If performed, finger thoracostomies or needle decompression should be followed by the insertion of chest drains with water seals. If prehospital thoracostomies (non-sterile) were performed, chest drains can be inserted using standard aseptic techniques in the previously performed thoracostomy or in novel thoracostomies according to local guidelines and practice.
- Tamponade and resuscitative thoracotomy: Immediate post-ROSC internal mammary and intercostal arteries severed due to the clamshell incision may start bleeding and may need clamping with small mosquito forceps. In all cases, patients should be facilitated without delay to the operating room. If a REBOA is in place, the inflation time should be kept to a minimum as feasible facilitating transfer to either operating room, angiography, or diagnostics as appropriate.

## Conclusions

The approach to TCA and traumatic peri-arrest states requires a structured approach based on treating all reversible causes simultaneously and rapidly. Multiple interventions need to be performed quickly requiring swift teamwork. Complexities of TCA management include rapid assessment and integration of information available and resultant rapid decision-making. Management of hypoxia, hypovolaemia, and release of tension pneumothorax or cardiac tamponade constitute the hallmark of treatment, with an eye on metabolic homeostasis constituting a more nuanced but fundamental approach. Advanced interventions include RT and REBOA which indications, timing, and limitations must be known by any provider dealing with TCA.

## Data Availability

Not applicable.

## Data availability

No datasets were generated or analysed during the current study.
